# Flavonoids from *Symplocos racemosa*

**DOI:** 10.3390/molecules20010358

**Published:** 2014-12-26

**Authors:** Mila Jung, Janggyoo Choi, Hee-Sung Chae, Jae Youl Cho, Young-Dong Kim, Khin Myo Htwe, Woo-Shin Lee, Young-Won Chin, Jinwoong Kim, Kee Dong Yoon

**Affiliations:** 1 College of Pharmacy, The Catholic University of Korea, Bucheon 420-743, Korea; E-Mail: jml333@nate.com; 2 College of Pharmacy and Research Institute of Pharmaceutical Science, Seoul National University, Seoul 151-742, Korea; E-Mails: dashutiao@naver.com (J.C.); jwkim@snu.ac.kr (J.K.); 3 College of Pharmacy and RFIND-BKplus Team, Dongguk University-Seoul, 32 Dongguk-lo, Ilsan dong-gu, Goyang, Gyeonggi-do 410-820, Korea; E-Mails: chaeheesung83@gmail.com (H.-S.C.); f2744@dongguk.edu (Y.-W.C.); 4 Department of Genetic Engineering, Sungkyunkwan University, Suwon 440-746, Korea; E-Mail: jaecho@skku.edu; 5 Department of Life Science, Hallym University, Chuncheon 200-702, Korea; E-Mail: ydkim@hallym.ac.kr; 6 Popa Mountain Park, Forest Department, Kyaukpadaung Township, Mandalay Division, Myanmar; E-Mail: khinmyohtwe007@gmail.com; 7 Department of Forest Sciences, Seoul National University, Seoul 151-921, Korea; E-Mail: krane@snu.ac.kr

**Keywords:** flavonoids, sympracemoside, *Symplocos racemosa*

## Abstract

A novel isoflavone glycoside, peseudobatigenin 7-*O*-[β-d-apiofuranosyl-(1''''→5''')-*O*-β-d-apiofuranosyl-(1'''→6'')]-β-d-glucopyranoside, namely sympracemoside (**1**), was isolated from the aerial parts of *Symplocos racemosa* along with 15 known flavonoids (**2**–**16**). Their structures were characterized by Q-TOF mass, optical rotation, UV, 1D and 2D-NMR spectroscopic data. Compounds **3**, **9**, **16** showed moderate inhibitory activities against NO production with IC_50_ value of 88.2, 42.1 and 74.3 μM, respectively.

## 1. Introduction

**Figure 1:**
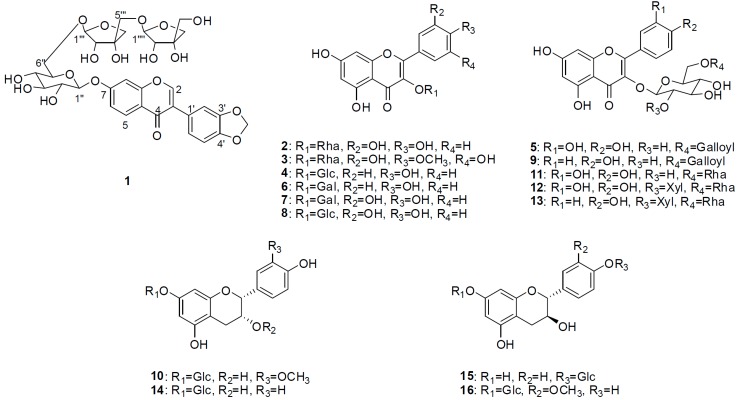
Chemical structures of compounds **1**–**16** from *Symplocos racemosa.*

*Symplocos racemosa* Roxb. is a small evergreen tree with a broad crown and stems grows up to 6 m high [1]. It belongs to the Symplocaceae family, which is a unigeneric family composed of only one genus, *i.e.*, *Symplocos*, and is distributed in tropical and subtropical regions of Asia, America, Australia and Malaysia [2]. In Myanmar, *S. racemosa* is called Dauk-yut or Mwet-kang, and its bark and stem have been used by local traditional practitioners to treat cough and fever. It is also well known as Lodhra in Ayurvedic remedies in India, and the astringent bark of this plant has been applied for the treatment of uterine disorders, diarrhea, dysentery, eye disease and menorrhagia as a single drug or an ingredient of multi-component preparations [3]. The phytochemical investigations of *S. racemosa* have mainly focused on the stems and barks, because these parts have long been used for medicinal purposes so far. The Pakistan research group exclusively reported phenolic glycosides and their biological activities from the bark of *S. racemosa* including salirepin, symplocuronic acid, sympocemoside [4], benzoyl salireposide, salireposide, four triterpenes, which featured phosphodiesterase inhibitory activity [5], symcomoside A and B, tortoside C, which showed α-glucosidase inhibitory acitivity, 1-ethyl brachiose-3'-acetate, nonaeicosanol and three fatty acid derivatives featuring lipoxygenase inhibitory acitivity [6], locoracemoside A, B, C describing α-chymotrypsin inhibitory activity [7]. Although phenolic glycosides and their biological activities have been well-reported so far, further phytochemical investigation of other constituents from *S. racemomsa* is required. The present study describes the isolation of 16 flavonoid derivatives, including a new compound, peseudobatigenin 7-*O*-[β-d-apiofuranosyl-(1''''→5''')-β-d-apiofuranosyl-(1'''→6'')]-β-d-glucopyranoside, namely sympracemoside (Figure 1), as well as their inhibitory activities against NO production.

## 2. Results and Discussion

### 2.1. Structural Elucidation of Isolated Compounds

**Figure 2:**
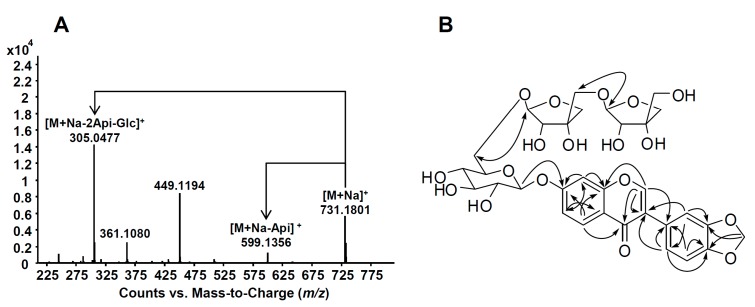
Q-TOF MS/MS (**A**) and HMBC correlations (**B**) of compound **1**.

Main text paragraph Compound **1** was isolated as a pale yellow amorphous powder, and Q-TOF MS revealed its molecular formula to be C_32_H_36_O_18_ from the pseudo-molecular ion peak at *m*/*z* 731.1801 (C_32_H_36_O_18_Na [M+Na]^+^). Figure 2A shows the MS/MS of **1** revealing [M+Na-Api]^+^ (*m*/*z* 599.1356) and [M+Na-2Api-Glc]^+^ (*m*/*z* 305.0477). The ^1^H-NMR spectrum of **1** showed a characteristic signal for the H-2 of isoflavone skeleton at δ 8.28 (1H, s, H-2), two sets of 1,3,4-trisubstituted aromatic rings at δ 8.16 (1H, d, *J* = 8.9 Hz, H-5), 7.28 (1H, d, *J* = 2.3 Hz, H-8), 7.23 (1H, dd, *J* = 8.9, 2.3 Hz, H-6), 7.09 (1H, d, *J* = 1.6 Hz, H-2'), 7.02 (1H, dd, *J* = 8.0, 1.6 Hz, H-6'), and 6.89 (1H, d, *J* = 8.0 Hz, H-5'), and the methylenedioxy group at δ 5.99 (2H, s). The methylenedioxy group (2H, δ_H_ 5.99) showed HMBC cross peaks at δ 149.2 (C-3') and 149.3 (C-4'), demonstrating the presence of 3',4'-methylenedioxy moiety. From a comparison of the ^1^H and ^13^C- NMR data for **1** with literature values, **1** was determined to be a pseudobaptigenin (7-hydroxy-3',4'-methylenedioxy- isoflavone) derivative [8]. Three anomeric protons arising from sugar moieties were detected in the ^1^H, ^13^C-NMR, and heteronuclear single quantum coherence (HSQC) at δ_H_ 5.07 (1H, d, *J* = 7.3 Hz)/δ_C_ 102.0 and δ_H_ 4.97 (2H, d, *J* = 2.6 Hz)/δ_C_ 111.0 (C×2) along with 13 oxygenated carbon signals, suggesting that **1** possessed one hexose and two pentose moieties. The ^13^C-NMR signals of the sugar moieties were in good agreement with previously published literature values for one glucopyranose and two apiofuranose groups [9]. The β-configuration of the glucopyranosyl and two apiopyranosyl moieties was assigned from the coupling constant of each anomeric proton in the ^1^H-NMR spectrum. The following inter-glycosidic linkages were established by exhaustive heteronuclear multiple bond correlation (HMBC) experiments (Figure 2B); H-1'''' of terminal Api (δ_H_ 4.97) to C-5''' of the middle Api (δ_C_ 71.8), H-1''' of the middle Api (δ_H_ 4.97) to C-6'' of Glc (δ_C_ 69.1) and H-1'' of Glc (δ_H_ 5.07) to C-7 of the pseudobaptigenin (δ_C_ 163.7). Therefore, the structure of **1** was determined to be peseudobatigenin 7-*O*-[β-d-apiofuranosyl-(1''''→5''')-β-d-apiofuranosyl-(1'''→6'')]-β-d-glucopyrano- side, namely sympracemoside.

The 15 known compounds that were isolated were identified as quercetin-3-*O*-α-l-rhamnopyranoside (**2**) [10], mearnsetin-3-*O*-α-l-rhamnopyranoside (**3**) [11], kaempferol-3-*O*-β-d-glucopyranoside (**4**) [10], quercetin-3-*O*-β-d-(6"-*O*-galloyl)-gulucopyranoside (**5**) [12], kaempferol-3-*O*-β-d-galactopyranoside (**6**) [10], quercetin-3-*O*-β-d-galactopyranoside (**7**) [10], quercetin-3-*O*-β-d-glucopyranoside (**8**) [10], kaempferol-3-*O*-β-d-(6"-*O*-galloyl)-gulucopyranoside (**9**) [12], 3'-*O*-methylepicatechin-7-*O*-β-d-glucopyranoside (**10**) [13], quercetin-3-*O*-rutinoside (**11**) [10], quercetin-3-*O*-(2*^G^*-β-d-xylopyranosylrutinoside) (**12**) [14], kaempferol-3-*O*-(2*^G^*-β-d-xylopyranosylrutinoside) (**13**) [15], (−)-epiafzelechin-7-*O*-β-d-glucopyranoside (**14**) [16], afzelechin-4'-*O*-β-d-glucopyranoside (**15**) [17] and 3'-*O*-methycatechin-7-*O*-β-d-glucopyranoside (**16**) [18] by comparing their spectroscopic data with those in literature.

*Sympracemoside* (**1**): pale yellowish amorphous powder, ${\left[\alpha \right]}_{\text{D}}^{25}$ −79.7° (*c* 0.1, MeOH); UV (MeOH) λ_max_ 248, 261, 291 nm; Q-TOF MS: *m/z* 731.1801 [M+Na]^+^ (calcd for C_32_H_36_O_18_Na 731.1799); ^1^H-NMR (CD_3_OD, 500 MHz): δ 3.51–3.71 (4H, m, H-2"-H-5"), 3.54 (1H, d, *J* = 9.9 Hz, H-5'''a), 3.54 (2H, m, H-5''''), 3.62 (1H, dd, *J* = 11.1, 7.1 Hz, H-6''a), 3.76 (1H, d, *J* = 9.7 Hz, H-4''''a), 3.76 (1H, d, *J* = 9.9 Hz, H-5'''b), 3.78 (1H, d, *J* = 9.7 Hz, H-4'''a), 3.93 (1H, d, *J* = 2.6 Hz, H-2''''), 3.96 (1H, d, *J* = 9.7 Hz, H-4''''b), 3.97 (1H, d, *J* = 2.6 Hz, H-2'''), 4.03 (1H, d, J = 9.7 Hz, H-4'''b), 4.07 (1H, dd, *J* = 11.1, 1.7 Hz, H-6''b), 4.97 (2H, d, *J* = 2.6 Hz, H-1''' and H-1''''), 5.07 (1H, d, *J* = 7.3 Hz, H-1"), 5.99 (2H, s, -O-CH_2_-O-, 6.89 (1H, d, *J* = 8.0 Hz, H-5'), 7.02 (1H, dd, *J* = 8.0, 1.6 Hz, H-6'), 7.09 (1H, d, *J* = 1.6 Hz, H-2'), 7.23 (1H, dd, *J* = 8.9, 2.3 Hz, H-6), 7.28 (1H, d, *J* = 2.3 Hz, H-8), 8.16 (1H, d, *J* = 8.9 Hz, H-5), 8.28 (1H, s, H-2); ^13^C-NMR (CD_3_OD-*d*_4_, 125 MHz): δ 65.5 (C-5''''), 69.1 (C-6''), 71.8 (C-5'''), 71.8 (C-4''), 74.9 (C-2''), 75.2 (C-4''''), 75.3 (C-4'''), 77.4 (C-5''), 78.0 (C-2''''), 78.1 (C-3''), 78.6 (C-2'''), 79.5 (C-3'''), 80.6 (C-3''''), 102.0 (C-1''), 102.7 (-O-CH_2_-O-), 105.2 (C-8), 109.4 (C-5'), 110.9 (C-2'), 111.0 (C-1'''), 111.0 (C-1''''), 117.4 (C-6), 120.4 (C-10), 123.9 (C-6'), 126.1 (C-3), 127.1 (C-1'), 128.5 (C-5), 149.2 (C-3'), 149.3 (C-4'), 155.7 (C-2), 159.4 (C-9), 163.7 (C-7), 178.0 (C-4).

### 2.2. Inhibitory Activity against NO Production

The inhibitory effects of compounds **1**–**16** against NO production were evaluated using lipopolysaccharide-induced RAW 264.7 cells. Among the tested compounds, mearnsetin-3-*O*-α-l-rhamnopyranoside (**3**), kaempferol-3-*O*-β-d-(6"-*O*-galloyl)-gulucopyranoside (**9**) and 3'-*O*-methycatechin-7-*O*-β-d-glucopyranoside (**16**) showed weak inhibitory activities against NO production with IC_50_ value of 88.2, 42.1 and 74.3 μM, respectively. The positive control, *i.e.*, dexamathasone, showed an IC_50_ value of 24.4 μM. The other compounds were found to be inactive against NO production in lipopolysaccharide-induced RAW 264.7 cells (Table 1).

**Table 1:** Effects of isolates on production of nitric oxide (NO) in lipopolysaccharide (LPS)-induced RAW 264.7 cells. The cells (1 × 10^5^ cells/mL) were pretreated with compounds 30 min prior to stimulation with LPS 24 hours after stimulation, the NO level of the supernatants was measured by Griess reagent.

Compounds	IC50 (µM)	Compounds	IC50 (µM)
**1**	100<	**9**	42.1
**2**	100<	**10**	100<
**3**	88.2	**11**	100<
**4**	100<	**12**	100<
**5**	100<	**13**	100<
**6**	100<	**14**	100<
**7**	100<	**15**	100<
**8**	100<	**16**	74.3

## 3. Experimental Section

### 3.1. General Experimental Procedures

^1^H-NMR and ^13^C-NMR spectra were recorded on a Bruker AscendTM 500 spectrometer. Mass spectra (Q-TOFMS) were obtained using an Agilent 6530 ESI-Q-TOF mass spectrometer. Optical rotations were recorded on a JASCO P-2000 polarimeter. UV spectra were measured using a Shimadzu UV-1800 spectrometer. The compounds were isolated using a Gilson preparative HPLC system (Gilson, USA) was applied to isolate compounds and equipped with binary pumps, an U*V*/*V*IS-155 detector, and an GX-271 liquid handler. The semi-preparative HPCCC instrument used in this study was a Spectrum (Dynamic Extractions, Berkshire, UK) combined to an IOTA S 300 pump (Ecom, Prague, Czech Republic), a Foxy R2 fraction collector (Teledyne Isco, NE, USA), and a CCA-1111 circulatory temperature regulator (Eyela, Tokyo, Japan).

Organic solvents for column chromatography were analytical grade and obtained from Daejung Chemical and Metals (Gyunggido, Korea). Acetonitrile, methanol, and water for HPLC were purchased from Fisher Scientific Korea (Seoul, Korea). Silica gel and reversed-phase silica gel were purchased from Merck (Germany), and Sephadex LH-20 was obtained from Pharmacia Co. (Sweden). HPLC was performed using an YMC-Pack ODS-A column (250 × 20 mm ID, 5 μm, Japan).

### 3.2. Plant Material

Aerial parts of *S. racemosa* were collected at Popa Mountain National Park (Mandalay, Myanmar) in August 2011, and identified by Prof. Young Dong Kim (Hallym University, Chuncheon, Korea). A voucher specimen (# MM-0097) was deposited at herbarium of National Institute of Biological Resources (NIBR) in Korea. 

### 3.3. Extraction and Isolation

The aerial parts of *Symplocos racemosa* (1012 g) were ground into fine powder, and extracted with methanol under ultrasoniation (3 × 3 h) to yield a methanol extract (52.7 g). The methanol extract was suspended in water and partitioned with *n*-hexane (7.0 g), ethyl acetate (12.3 g), and *n*-butanol (9.1 g). The ethyl-acetate fraction was subjected to silica-gel column chromatography (CC) with a step gradient elution of chloroform-methanol (20:1 to 1:1, *v*/*v*) to obtain four subfractions (fractions E1–E4). Fraction E3 (2.2 g) was chromatographed on Sephadex LH-20 CC with a methanol to yield four subfractions (Fractions E3-1–E3-4), and E3-1 was further subjected to reversed-phase (RP) HPLC using an acetonitrile-water mixture (30:70 *v*/*v*) to give quercetin-3-*O*-α-l-rhamnopyranoside (1.6 mg) and mearnsetin-3-*O*-α-l-rhamnopyranoside (8.0 mg). Kaempferol-3-*O*-β-d-glucopyranoside, (22 mg) and quercetin-3-*O*-β-d-(6"-*O*-galloyl)-glucopyranoside (5.5 mg) were isolated from E3-2 (226 mg) through Sephadex LH-20 CC with methanol followed by repetitive RP- HPLC using acetonitrile-water mixture (30:70 *v*/*v*). Fraction E3-3 (610 mg) were subjected to RP-HPLC (acetonitrile:water = 30:70, *v*/*v*) to give kaempferol-3-*O*-β-d-galactopyranoside (1.9 mg), quercetin-3-*O*-β-d-galactopyranoside (11.9 mg) and quercetin-3-*O*-β-d-glucopyranoside (16.4 mg). Kaempferol-3-*O*-β-d-(6"-*O*-galloyl)-glucopyranoside (2.9 mg) was separated from E4 via Sephadex LH-20 column CC using methanol as the mobile phase, followed by RP-HPLC (methanol-water, 52:48, *v*/*v*).

The *n*-butanol (9 g) soluble fraction was subjected to Diaion HP-20 CC to give two fractions [water fraction (BW) and methanol fraction (BM)]. BM (2.7 g) was chromatographed using high-performance countercurrent chromatography (HPCCC) with ethyl acetate–*n*-butanol–water system (3:7:10, *v*/*v*, 1500 rpm, 15 mL/min, lower phase was used as a mobile phase) to give fraction BM1–BM4. BM2 (208 mg) was separated by Sephadex LH-20 CC using methanol as an eluent followed by RP-HPLC to yield 3'-*O*-methylepicatechin-7-*O*-β-d-glucopyranoside (5.9 mg), 3'-*O*-methycatechin-7-*O*-β-d-glucopyranoside (1.6 mg), quercetin-3-*O*-rutinoside (14.4 mg) and quercetin-3-*O*-(2*^G^*-β-d-xylopyranosylrutinoside (20.2 mg). BM3 (123 mg) was subjected to RP-HPLC using a gradient elution of acetonitrile-water mixture (15%→30% acetonitrile) to yield (−)-epiafzelechin-7-*O*-β-d-glucopyranoside (2.4 mg), afzelechin-4'-*O*-β-d-glucopyranoside (1.3 mg), sympracemoside (2.3 mg).

### 3.4. Measurement of NO Production

NO production was assayed by measuring the amount of nitrite in the supernatants of cultured RAW 264.7 cells. Brieﬂy, the cells were seeded at a density of 5 × 10^5^ cells/mL in 96-well culture plates. After pre-incubation for 18 h, the cells were pretreated for 30 min with compounds and then stimulated with LPS (500 ng/mL) for 24 h. The supernatant was mixed with an equal volume of Griess reagent (1% sulfanilamide, 0.1% naphthylethylenediamine dihydrochloride, and 2.5% phosphoric acid) and then incubated at room temperature for 5 min. The concentration of nitrite was determined by measuring the absorbance at 570 nm and comparing the values to a standard curve generated using sodium nitrite (NaNO_2_).

## Supplementary Materials

Supplementary materials can be accessed at: http://www.mdpi.com/1420-3049/20/01/00358/s1.
