# Supplementary material for: Flavonoids from Symplocos racemosa
Source: Molecules. 2014 Dec 26;20(1):358–65. doi: 10.3390/molecules20010358 (PMC6272286; doi:10.3390/molecules20010358)
Supplement: Supplementary file 1 [file molecules-20-00358-s001.pdf]

Supplementary Materials

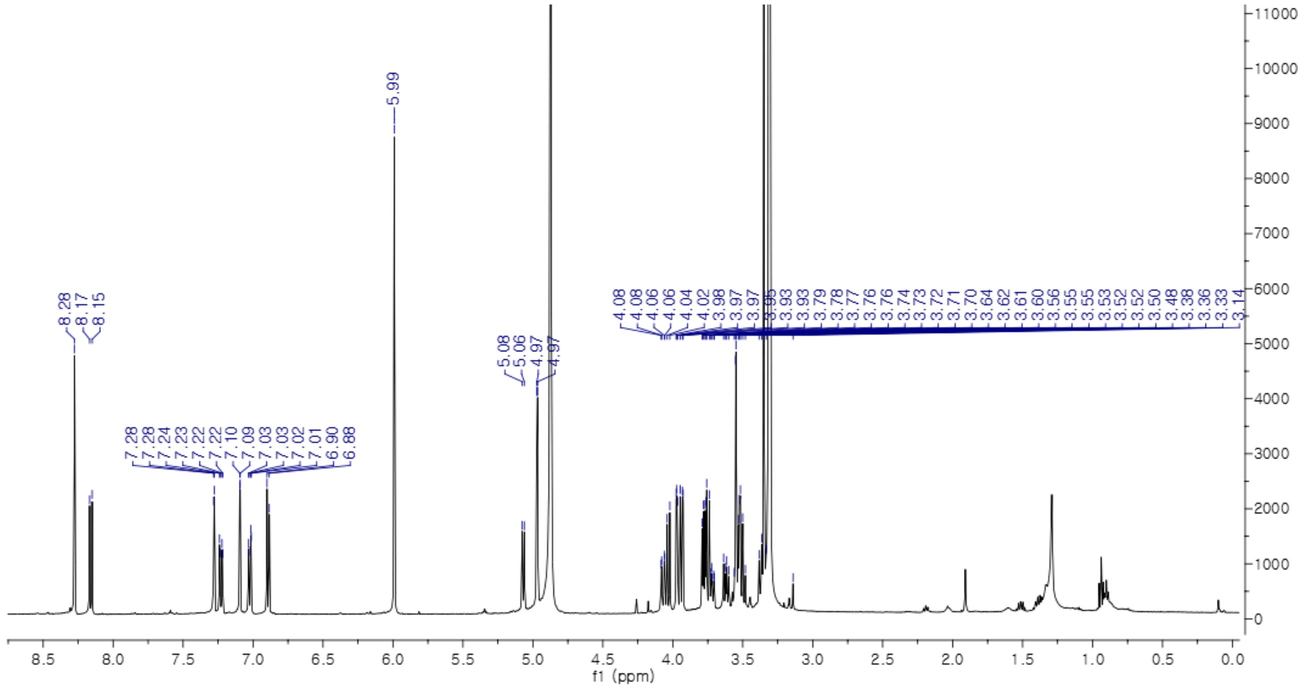

Figure S1. ^{1}H-NMR spectrum of sympracemoside (**1**) (CD_3OD, 500 MHz, δ ppm).

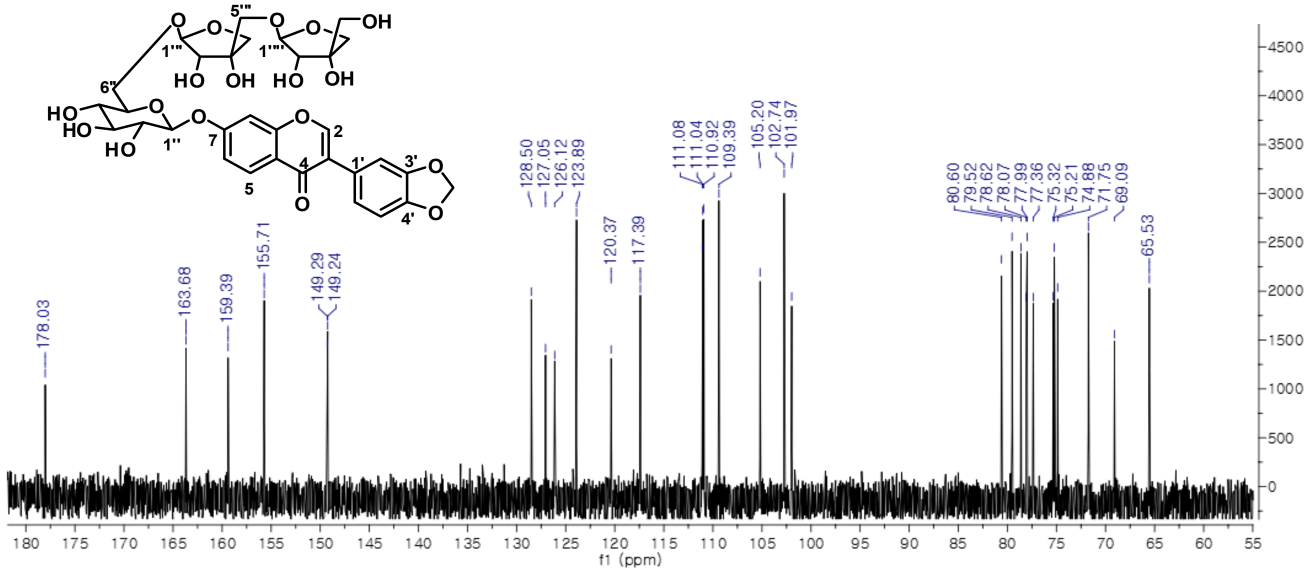

Figure S2. ^{13}C-NMR spectrum of sympracemoside (**1**) (CD_3OD, 125 MHz, δ ppm).

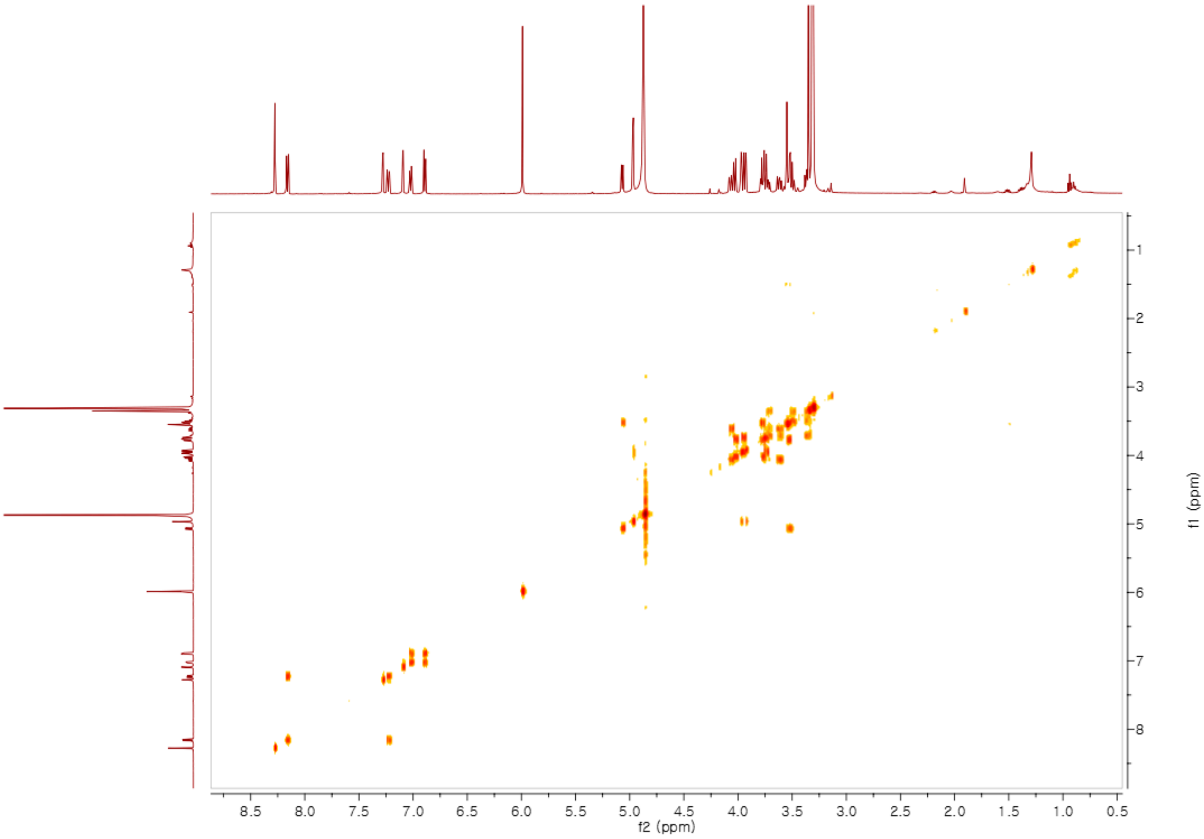

Figure S3. ^{1}H-^{1}H COSY spectrum of sympracemoside (**1**) (CD_3OD, δ ppm).

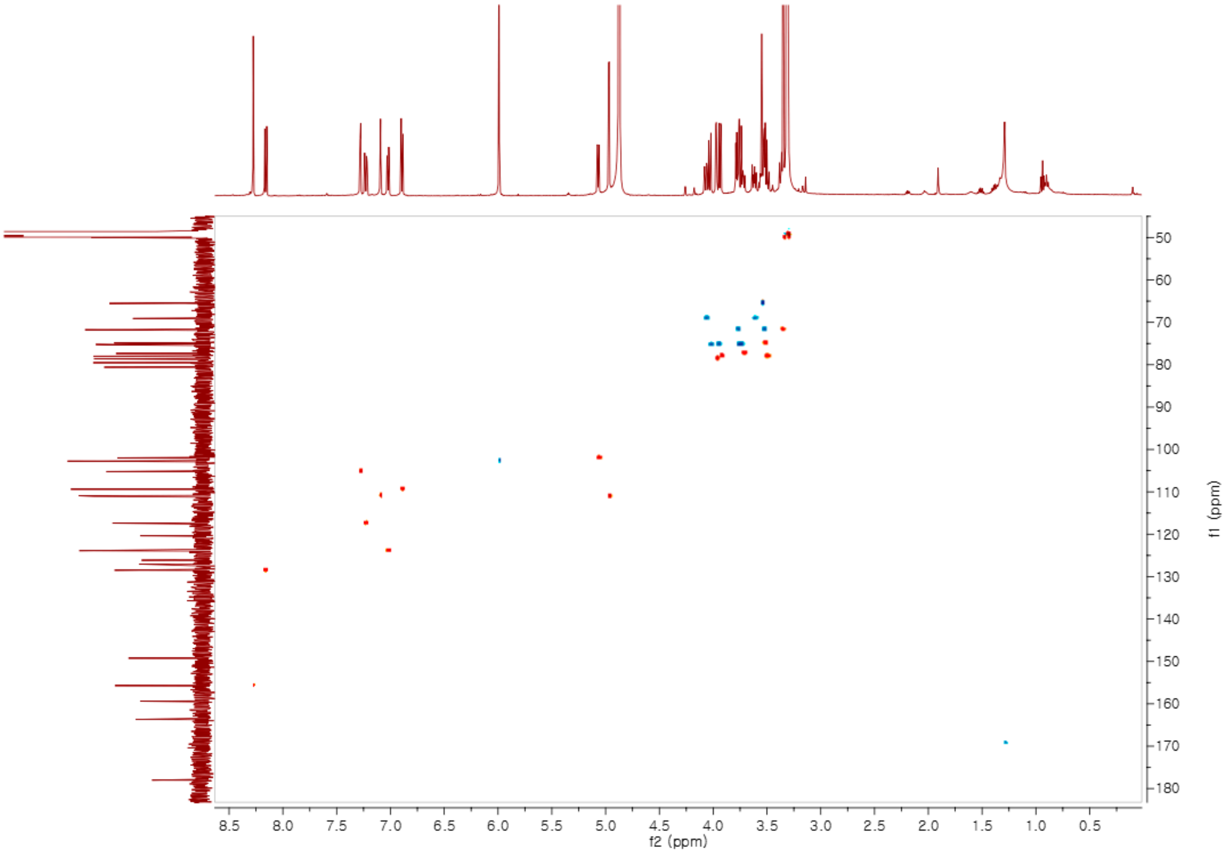

Figure S4. HSQC spectrum of sympracemoside (**1**) (CD_3OD, δ ppm).

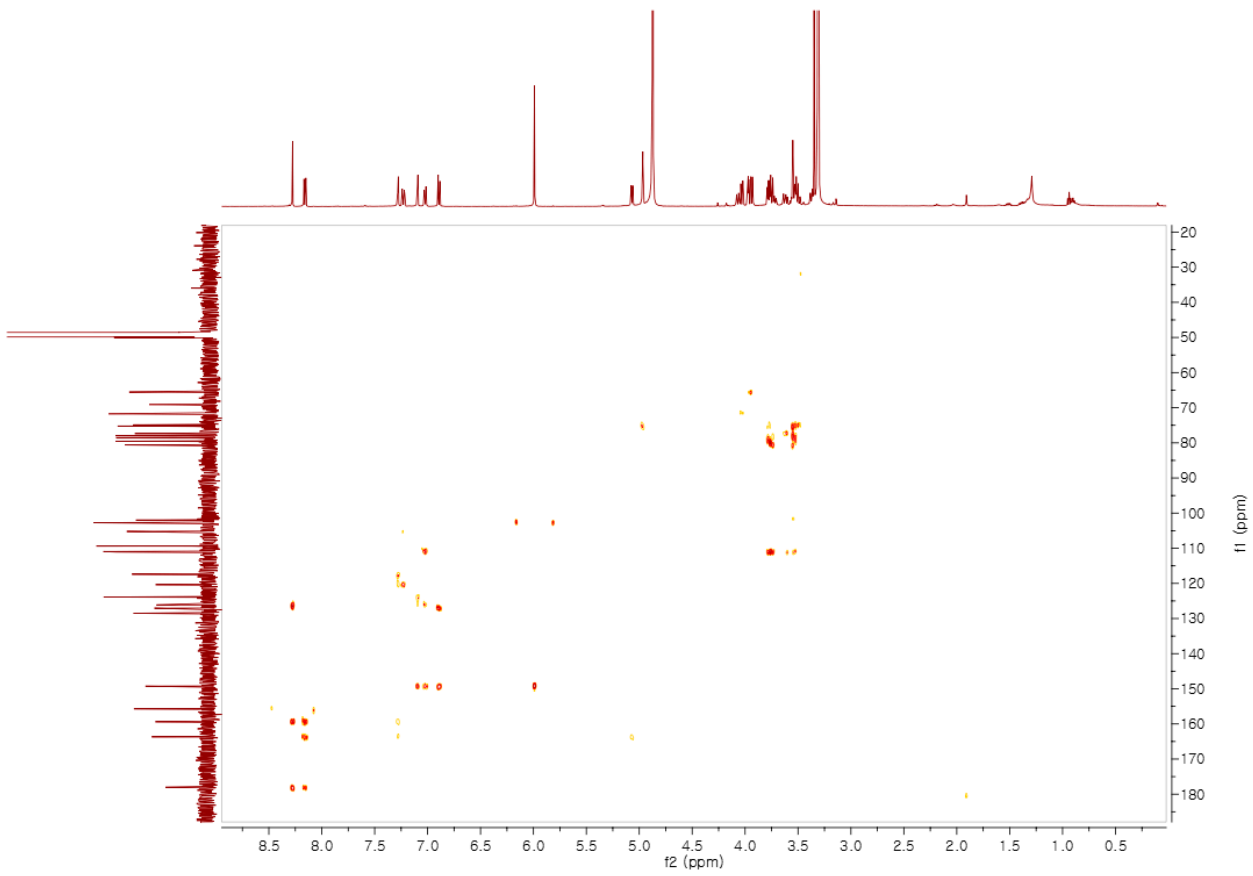

Figure S5. HMBC spectrum of sympracemoside (**1**) (CD_3OD, δ ppm).

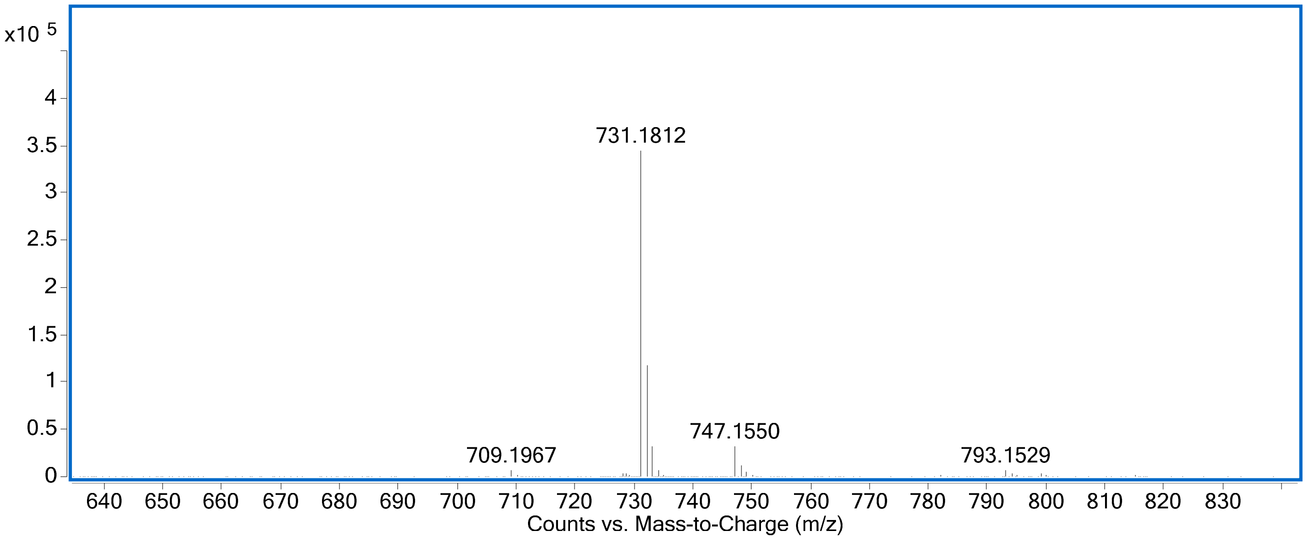

Figure S6. Q-TOF MS of sympracemoside (**1**).

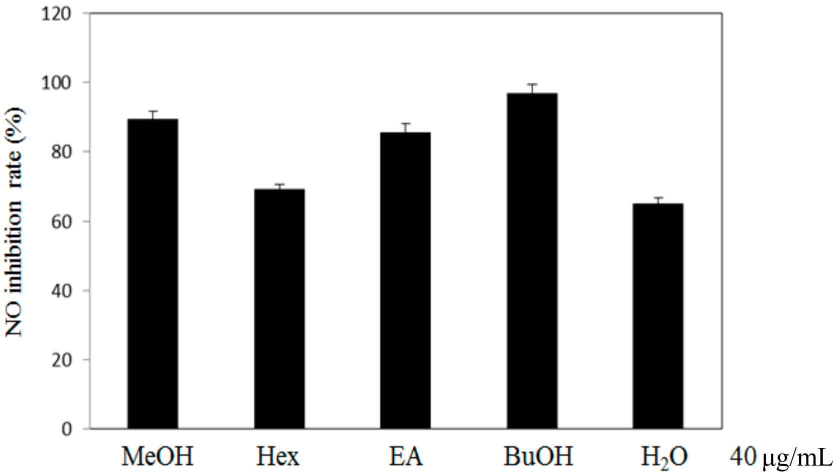

Figure S7. The Effects of the MeOH, Hex, EA, BuOH and H_2O Fraction of *S. racemosa* on Nitric oxide inhibition rate in LPS induced RAW 264.7 cells.

Spectroscopic Data of Compounds 2–16

Quercetin-3-O-α-L-rhamnopyranoside (**2**): yellow amorphous powder; Q-TOF MS: *m/z* 449.1087 $[M+H]^+$ (calcd for $C_{21}H_{21}O_{11}$ 449.1084); ^{1}H-NMR (500 MHz, DMSO-d_6): δ 0.82 (3H, d, *J* = 6.1 Hz, H-6''), 3.13–3.97 (4H, m, H-2''- 5''), 5.25 (1H, d, *J* = 1.4 Hz, H-1''), 6.16 (1H, s, H-6), 6.34 (1H, s, H-8), 6.85 (1H, d, *J* = 8.3 Hz, H-6'), 7.25 (1H, dd, *J* = 8.3, 2.2 Hz, H-5'), 7.29 (1H, d, *J* = 2.2 Hz, H-2'); ^{13}C-NMR (125 MHz, DMSO-d_6): δ 17.4 (C-6''), 70.0 (C-5''), 70.3 (C-2''), 70.6 (C-4''), 71.2 (C-3''), 93.6 (C-8), 98.7 (C-6), 101.8 (C-1''), 104.1 (C-10), 115.4 (C-5'), 115.6 (C-2'), 120.7 (C-1'), 121.0 (C-6'), 134.2 (C-3), 145.2 (C-4'), 148.4 (C-3'), 156.4 (C-9), 158.3 (C-2), 161.2 (C-5), 163.2 (C-7), 177.5 (C-4).

Mearnsetin-3-O-α-L-rhamnopyranoside (**3**): yellow amorphous powder; Q-TOF MS: *m/z* 479.1196 $[M+H]^+$ (calcd for $C_{22}H_{23}O_{12}$ 479.1190); ^{1}H-NMR (500 MHz, DMSO-d_6): δ 0.81 (3H, d, *J* = 5.7 Hz, H-6''), 3.14–3.98 (4H, m, H-2''- 5''), 3.74 (3H, s, 4'-OCH_3), 5.15 (1H, d, *J* = 1.4 Hz, H-1''), 6.19 (1H, d, *J* = 1.9 Hz, H-6), 6.35 (1H, d, *J* = 1.9 Hz, H-8), 6.82 (2H, s, H-2', 6'); ^{13}C-NMR (125 MHz, DMSO-d_6): δ 17.4 (C-6''), 59.7 (-O$\underline{C}H_3$), 70.0 (C-2''), 70.3 (C-5''), 70.5 (C-3''), 71.1 (C-4''), 93.6 (C-8), 98.8 (C-6), 102.1 (C-1''), 104.0 (C-10), 108.1 (C-2', 6'), 124.8 (C-1'), 134.8 (C-3), 137.7 (C-4'), 150.6 (C-3', 5'), 156.5 (C-9), 157.1 (C-2), 161.2 (C-5), 164.7 (C-7), 177.8 (C-4).

Kaempferol-3-O-β-D-glucopyranoside (**4**): yellow amorphous powder; Q-TOF MS: *m/z* 449.1092 $[M+H]^+$ (calcd for $C_{21}H_{21}O_{11}$ 449.1084); ^{1}H-NMR (500 MHz, DMSO-d_6): δ 3.06–3.56 (6H, m, H-2''-6''), 5.45 (1H, d, *J* = 7.5 Hz, H-1''), 6.19 (1H, d, *J* = 2.0 Hz, H-6), 6.41 (1H, d, *J* = 2.0 Hz, H-8), 6.88 (2H, d, *J* = 8.8 Hz, H-2', 6'), 8.03 (2H, d, *J* = 8.8 Hz, H-3', 5'); ^{13}C-NMR (125 MHz, DMSO-d_6): δ 60.8 (C-6''), 69.8 (C-4''), 74.1 (C-2''), 76.4 (C-3''), 77.4 (C-5''), 93.6 (C-8), 98.7 (C-6), 100.8 (C-1''), 103.8 (C-10), 115.0 (C-3'), 120.8 (C-1'), 130.8 (C-2'), 133.1 (C-3), 153.2 (C-5), 156.3 (C-9), 159.9 (C-4'), 161.1 (C-2), 164.8 (C-7), 177.3 (C-4).

Quercetin-3-O-(6β-O-galloyl-β-D-glucopyranoside) (**5**): yellow amorphous powder; Q-TOF MS: *m/z* 617.1150 [M+H]$^+$ (calcd for $C_{28}H_{25}O_{16}$ 617.1143); ^{1}H-NMR (500 MHz, DMSO-d_6): δ 3.27–4.25 (6H, m, H-2''- 6''), 5.44 (1H, d, J = 7.4 Hz, H-1''), 6.17 (1H, d, J = 2.0 Hz, H-6), 6.36 (1H, d, J = 2.0 Hz, H-8), 6.72 (1H, d, J = 8.4 Hz, H-5'), 6.89 (2H, s, H-2''', 6'''), 7.43 (1H, d, J = 2.2 Hz, H-2'), 7.57 (1H, dd, J = 8.4, 2.2 Hz, H-6'); ^{13}C-NMR (125 MHz, DMSO-d_6) δ: 63.1 (C-6''), 69.4 (C-4''), 74.0 (C-2''), 74.2 (C-3''), 76.2 (C-5''), 93.5 (C-8), 98.7 (C-6), 101.4 (C-1''), 103.8 (C-10), 108.5 (C-2''', 6'''), 115.2 (C-3'), 115.7 (C-5'), 119.2 (C-1'''), 120.8 (C-1'), 121.8 (C-6'), 133.3 (C-3), 138.4 (C-4'''), 144.8 (C-3'), 145.4 (C-2''', 6'''), 148.6 (C-4'), 156.2 (C-9), 156.4 (C-2), 161.1 (C-5), 164.2 (C-7), 165.6 (C-7'''), 177.2 (C-4).

Kaempferol-3-O-β-D-galactopyranoside (**6**): yellow amorphous powder; Q-TOF MS: *m/z* 471.0906 [M+Na]$^+$ (calcd for $C_{21}H_{20}O_{11}Na$ 471.0903); ^{1}H-NMR (500 MHz, DMSO-d_6): δ 3.06–3.56 (6H, m, H-2''- 6''), 5.45 (1H, d, J = 7.5 Hz, H-1''), 6.19 (1H, d, J = 2.0 Hz, H-6), 6.41 (1H, d, J = 2.0 Hz, H-8), 6.88 (2H, d, J = 8.8 Hz, H-2', 6'), 8.03 (2H, d, J = 8.8 Hz, H-3', 5'); ^{13}C-NMR (125 MHz, DMSO-d_6): δ 60.0 (C-6''), 67.8 (C-4''), 71.2 (C-2''), 73.1 (C-3''), 75.7 (C-5''), 93.7 (C-8), 98.9 (C-6), 102.3 (C-1''), 103.8 (C-10), 114.9 (C-3'), 121.0 (C-1'), 130.7 (C-2'), 133.0 (C-3), 156.6 (C-5), 156.8 (C-9), 161.7 (C-4'), 164.1 (C-2), 164.1 (C-7), 177.3 (C-4).

Quercetin-3-O-β-D-galactopyranoside (**7**): yellow amorphous powder; Q-TOF MS: *m/z* 465.1040 [M+H]$^+$ (calcd for $C_{21}H_{21}O_{12}$ 465.1033); ^{1}H-NMR (500 MHz, DMSO-d_6): δ 3.21–3.64 (6H, m, H-2''- 6''), 5.37 (1H, d, J = 7.7 Hz, H-1''), 6.17 (1H, s, H-6), 6.38 (1H, s, H-8), 6.81 (1H, d, J = 8.4 Hz, H-2'), 7.52 (1H, d, J = 1.8 Hz, H-5'), 7.66 (1H, dd, J = 8.4, 1.8 Hz, H-6'); ^{13}C-NMR (125 MHz, DMSO-d_6): δ 60.2 (C-6''), 68.0 (C-4''), 71.3 (C-2''), 73.2 (C-3''), 75.9 (C-5''), 93.6 (C-8), 98.8 (C-6), 101.0 (C-1''), 103.9 (C-10), 115.3 (C-5'), 116.0 (C-2'), 121.2 (C-1'), 122.0 (C-6'), 133.5 (C-3), 144.8 (C-4'), 156.2 (C-2), 156.4 (C-9), 161.3 (C-5), 164.3 (C-7), 177.5 (C-4).

Quercetin-3-O-β-D-glucopyranoside (**8**): yellow amorphous powder; Q-TOF MS: *m/z* 465.1044 [M+H]$^+$ (calcd for $C_{21}H_{21}O_{12}$ 465.1033); ^{1}H-NMR (500 MHz, DMSO-d_6): δ 3.08–3.59 (6H, m, H-2''- 6''), 5.45 (1H, d, J = 7.5 Hz, H-1''), 6.17 (1H, d, J = 2.0 Hz, H-6), 6.37 (1H, d, J = 2.0 Hz, H-8), 6.83 (1H, d, J = 9.0 Hz, H-5'), 7.57 (1H, dd, J = 9.0, 2.2 Hz, H-6'), 7.57 (1H, d, J = 2.2 Hz, H-2'); ^{13}C-NMR (125 MHz, DMSO-d_6): δ 61.0(C-6''), 69.9 (C-4''), 74.1 (C-2''), 76.5 (C-3''), 77.6 (C-5''), 93.6 (C-8), 98.8 (C-6), 100.9 (C-1''), 103.8 (C-10), 115.2 (C-2'),116.2 (C-5'), 121.1 (C-1'), 121.6 (C-6'), 133.3 (C-3), 144.8 (C-3'), 148.5 (C-4'), 156.1 (C-2),156.4 (C-9),161.2 (C-5), 164.6 (C-7), 177.4 (C-4).

Kaempferol-3-O-(6β-O-galloyl-β-D-glucopyranoside) (**9**): yellow amorphous powder; Q-TOF MS: *m/z* 601.1202 [M+H]$^+$ (calcd $C_{28}H_{25}O_{15}$ for 617.1193); ^{1}H-NMR (500 MHz, DMSO-d_6): δ 3.16–4.26 (6H, m, H-2''- 6''), 5.44 (1H, d, J = 7.5 Hz, H-1''), 6.19 (1H, d, J = 1.8 Hz, H-6), 6.39 (1H, d, J = 1.8 Hz, H-8), 6.76 (2H, d, J = 8.8 Hz, H-3', 5'), 6.91 (2H, s, H-2''', 6'''), 7.93 (2H, d, J = 8.8 Hz, H-2', 6'); ^{13}C-NMR (125 MHz, DMSO-d_6): δ 62.7 (C-6''), 69.2 (C-4''), 74.0 (C-3''), 74.0 (C-2''), 76.1 (C-5''), 93.7 (C-8), 98.7 (C-6), 101.4 (C-1''), 103.9 (C-10), 108.5 (C-2''', 6'''), 115.0 (C-3', 5'), 119.2 (C-1'''), 120.6 (C-1'), 130.7 (C-2', 6'), 133.1 (C-3), 138.3 (C-4'''), 145.4 (C-3''', 5'''), 156.3 (C-9), 156.7 (C-2), 159.8 (C-4'), 161.1 (C-5), 164.1 (C-7), 165.6 (C-7'''), 177.2 (C-4).

*3'-O-Methylepicatechin-7-O-b-*D*-glucopyranoside* (**10**): Q-TOF MS: *m/z* 489.1379 [M+Na]$^+$ (calcd $C_{22}H_{26}O_{11}Na$ for 489.1373); ^{1}H-NMR (CD_3OD, 500 MHz): δ 2.91 (1H, dd, 17.0, 4.6 Hz, H-4a), 2.97 (1H, dd, *J* = 17.0, 2.5 Hz, H-4b), 3.73 (1H, m, H-3), 3.87 (3H, s, 3'-OCH_3), 4.19 (1H, brs, H-2), 4.85 (1H, d, *J* = 7.9 Hz, H-1''), 6.09 (1H, d, *J* = 2.2 Hz, H-6), 6.29 (1H, d, *J* = 2.2 Hz, H-8), 6.79 (1H, d, *J* = 8.2 Hz, H-5'), 6.91 (1H, dd, *J* = 8.2, 1.7 Hz, H-6'), 7.13 (1H, d, *J* = 1.7 Hz, H-2'); ^{13}C-NMR (DMSO-d_6, 125 MHz): 28.4 (C-4), 55.6 (-O<u>C</u>H_3), 60.6 (C-6''), 64.6 (C-3), 69.6 (C-4''), 73.3 (C-2''), 76.7(C-3''), 76.9(C-5''), 78.2 (C-2), 95.2 (C-8), 96.5 (C-6), 100.7 (C-1''), 100.8 (C-10), 111.6 (C-2'), 114.7 (C-5'), 119.6 (C-6'), 130.5 (C-1'), 145.8 (C-4'), 146.9 (C-3'), 155.3 (C-5), 156.4 (C-7), 156.8 (C-9).

Quercetin-3-O-rutinoside (**11**): Q-TOF MS: *m/z* 611.1620 [M+H]$^+$ (calcd $C_{27}H_{31}O_{16}$ for 611.1612); ^{1}H-NMR (DMSO-d_6, 500 MHz): d 0.99 (3H, d, *J* = 6.1 Hz, H-6'''), 4.38 (1H, s, H-1'''), 5.33 (1H, d, *J* = 7.0 Hz, H-1''), 6.17 (1H, brs, H-6), 6.36 (1H, brs, H-8), 6.83 (1H, d, *J* = 8.2 Hz, H-5'), 7.53 (1H, dd, *J* = 8.2, 1.9 Hz, H-6'), 7.55 (1H, d, *J* = 1.9 Hz, H-2'); ^{13}C-NMR (DMSO-d_6, 125 MHz): d 17.7 (C-6'''), 48.6 (C-6''), 67.0 (C-5'''), 68.2 (C-2'''), 70.0 (C-3'''), 70.3 (C-4'''), 70.5 (C-4''), 71.8 (C-2''), 74.1 (C-5''), 75.9 (C-3''), 76.4 (C-8), 93.6 (C-6), 98.7 (C-1''), 100.7 (C-1'''), 101.2 (C-10), 103.8 (C-2'), 115.2 (C-5'), 116.2 (C-6'), 121.1 (C-1'), 121.6 (C-3), 133.3 (C-3'), 144.8 (C-4'), 148.5 (C-2), 156.4 (C-5), 156.5 (C-9), 161.2 (C-7), 177.3 (C-4).

*Quercetin-3-O-(2^G-b-*D*-xylopyranosylrutinoside)* (**12**): Q-TOF MS: *m/z* 743.2043 [M+H]$^+$ (calcd $C_{32}H_{39}O_{20}$ for 743.2035); ^{1}H-NMR (DMSO-d_6, 500 MHz): d 0.96 (3H, d, *J* = 6.2 Hz, H-6'''), 4.35 (1H, s, H-1'''), 4.57 (1H, d, *J* = 7.2 Hz, H-1''''), 5.61 (1H, d, *J* = 7.0 Hz, H-1''), 6.17 (1H, d, *J* = 1.2 Hz, H-6), 6.37 (1H, d, *J* = 1.2 Hz, H-8), 6.84 (1H, d, *J* = 8.5 Hz, H-5'), 7.52 (1H, d, *J* = 2.1 Hz, H-2'), 7.60 (1H, dd, *J* = 8.5, 2.1 Hz, H-6'); ^{13}C-NMR (DMSO-d_6, 125 MHz): d 17.7 (C-6'''), 65.6 (C-5''''), 66.4 (C-6''), 68.2 (C-5'''), 69.4 (C-4''''), 69.6 (C-3'''), 70.3 (C-2'''), 70.5 (C-4''), 71.8 (C-4'''), 73.8 (C-2''''), 75.9 (C-3''''), 76.0 (C-5''), 76.7 (C-3''), 81.6 (C-2''), 93.5 (C-8), 98.2 (C-6), 98.6 (C-1''), 100.5 (C-1'''), 103.8 (C-10), 104.4 (C-1''''), 115.3 (C-2'), 116.1 (C-5'), 121.2 (C-1'), 121.8 (C-6'), 132.9 (C-3), 144.8 (C-3'), 148.4 (C-4'), 155.8 (C-9), 156.3 (C-2), 161.2 (C-5), 164.1 (C-7), 177.3(C-4)

*Kaempferol-3-O-(2^G-b-*D*-xylopyranosylrutinoside)* (**13**): Q-TOF MS: *m/z* 727.2088 [M+H]$^+$ (calcd $C_{32}H_{39}O_{19}$ for 727.2086); ^{1}H NMR (DMSO-d_6, 500 MHz): d 4.34 (1H, s, H-1''''), 4.59 (1H, d, *J* = 7.2 Hz, H-1'''), 5.56 (1H, d, *J* = 7.3 Hz, H-1''), 6.07 (1H, s, H-6), 6.27 (1H, s, H-8), 6.87 (2H, d, *J* = 8.8 Hz, H-3', 5'), 8.00 (2H, d, *J* = 8.8 Hz, H-2',6'); ^{13}C-NMR (CD_3OD, 500 MHz): d 18.0 (C-6''''), 66.7 (C-5'''), 68.3 (C-6''), 69.9 (C-5''''), 71.2 (C-4'''), 71.6 (C-4''), 72.2 (C-2''''), 72.4 (C-3''''), 74.0 (C-4''''), 74.9 (C-2'''), 77.0 (C-5''), 77.3 (C-3'''), 78.4 (C-3''), 82.2 (C-2''), 95.0 (C-8), 101.0 (C-6), 102.3 (C-1''''), 105.4 (C-10), 105.8 (C-1''),105.8 (C-1'''), 116.3 (C-3', 5'), 123.1 (C-1'), 132.5 (C-2', 6'), 134.9 (C-3), 158.7 (C-2), 158.8 (C-9), 161.6 (C-4'), 163.4 (C-5), 166.3 (C-7), 179.6 (C-4)

*(-)-Epiafzelechin-7-O-b-*D*-glucopyranoside* (**14**): Q-TOF MS: *m/z* 459.1268 [M+Na]$^+$ (calcd $C_{21}H_{24}O_{10}Na$ for 459.1267); ^{1}H NMR (CD_3OD, 500 MHz): d 2.90 (1H, d, *J* = 16.0, 2.0 Hz, H-4a), 2.96 (1H, d, *J* = 16.0, 3.0 Hz, H-4b), 3.72 (1H, dd, *J* = 4.9. 11.8 Hz, H-6''a), 3.91 (1H, dd, J = 11.2, 1.5 Hz, H-6''b), 4.18 (1H, m, H-3), 4.85 (1H, d, *J* = 7.69 Hz, H-1''), 4.85 (1H, s, H-2), 6.08 (1H, d, *J* = 2.2 Hz, H-6), 6.28 (1H, d, *J* = 2.2 Hz, H-8), 6.78 (2H, d, *J* = 8.6 Hz, H-3', 5'), 7.32 (2H, d, *J* = 8.6 Hz, H-2', 6'); ^{13}C NMR (CD_3OD, 125 MHz): d 29.5 (C-4), 29.5 (C-6''), 62.7 (C-3), 67.4 (C-4''), 71.5 (C-2''), 75.1

(C-5''), 78.3 (C-3''),78.4 (C-2), 79.9 (C-8), 80.2 (C-6), 97.1 (C-1''), 98.6 (C-10), 102.7 (C-3', 5'), 115.9 (C-2', 6'), 129.3 (C-1'), 131.6 (C-9), 157.3 (C-5), 158.0 (C-9), 158.1 (C-4'), 158.6 (C-7).

Afzelechin-4'-O-b-D-glucopyranoside (**15**): Q-TOF MS: *m/z* 459.1262 $[M+Na]^+$ (calcd $C_{21}H_{24}O_{10}Na$ for 459.1267); ^{1}H-NMR (CD_3OD, 500 MHz): d 2.56 (1H, dd, *J* = 16.4, 8.5 Hz, H-4'), 3.06 (1H, dd, *J* = 16.4, 5.4 Hz, H-4), 3.99 (1H, m, H-3), 4.62 (1H, d, *J* = 7.7 Hz, H-2), 4.84 (1H, d, *J* = 7.5 Hz, H-1''), 6.01 (1H, d, *J* = 2.2 Hz, H-6) , 6.27 (1H, d, *J* = 2.2 Hz, H-8), 6.78 (2H, d, *J* = 8.6 Hz, H-3', 5'), 7.21 (2H, d, *J* = 8.6 Hz, H-2', 6'); ^{13}C-NMR (DMSO-d_6, 125 MHz): d 29.0 (C-4), 60.6 (C-6'').66.1 (C-3), 69.6 (C-5''), 73.2 (C-2''), 77.0 (C-4''), 76.7 (C-3''), 81.1 (C-2), 95.0 (C-8), 96.1 (C-6), 100.6 (C-10), 101.3 (C-1''), 114.8 (C-3', 5'), 132.5 (C-1'), 128.6 (C-2', 6'), 155.0 (C-9), 156.7 (C-7), 155.4 (C-5), 157.0 (C-4').

3'-O-Methycatechin-7-O-b-D-glucopyranoside ((**16**): Q-TOF MS: *m/z* 489.1377 $[M+Na]^+$ (calcd $C_{22}H_{26}O_{11}Na$ for 489.1373); ^{1}H NMR (CD_3OD, 500 MHz): d 2.57 (1H, dd, *J* = 16.4, 8.5 Hz, H-4a), 3.07 (1H, dd, *J* = 16.4, 5.4 Hz, H-4b), 3.72 (1H, dd, *J* = 11.8, 2.0 Hz, H-6''a), 3.85 (3H, s, 3'-OCH_3), 3.91 (1H, brd, *J* = 11.8 Hz, H-6''b), 4.01 (1H, m, H-3), 4.61 (1H, d, *J* = 7.8 Hz, H-2), 4.85 (1H, d, *J* = 7.6 Hz, H-1''), 6.02 (1H, d, *J* = 2.2 Hz, H-6), 6.27 (1H, d, *J* = 2.2 Hz, H-8), 6.79 (1H, d, *J* = 8.0 Hz, H-5'), 6.84 (1H, dd, *J* = 8.0, 1.5 Hz, H-6'), 6.96 (1H, d, *J* = 1.5 Hz, H-2'); ^{13}C NMR (CD_3OD, 125 MHz): d 29.0 (C-4), 56.5 (-O$\underline{C}H_3$), 62.7 (C-6''), 68.8 (C-3), 71.5 (C-4''), 75.0 (C-2''), 78.3 (C-3''), 78.4 (C-5''), 83.3 (C-2), 97.1 (C-6), 98.2 (C-8), 102.7 (C-1''), 103.6 (C-10), 112.0 (C-2'), 116.1 (C-6'), 121.5 (C-5'), 132.1 (C-1'), 147.7 (C-4'), 149.1 (C-3'), 156.9 (C-9), 158.1 (C-7), 158.3 (C-5).
